# Bacterial toxin modulation of the eukaryotic cell cycle: are all cytolethal distending toxins created equally?

**DOI:** 10.3389/fcimb.2012.00124

**Published:** 2012-10-08

**Authors:** Amandeep Gargi, Michael Reno, Steven R. Blanke

**Affiliations:** Department of Microbiology, Institute for Genomic Biology, University of IllinoisUrbana, IL, USA

**Keywords:** cytolethal distending toxin, CDT, cell cycle arrest, bacterial toxin, genotoxin, cyclomodulin

## Abstract

The cytolethal distending toxins (CDTs) comprise a family of intracellular-acting bacterial protein toxins whose actions upon eukaryotic cells result in several consequences, the most characteristic of which is the induction of G_2_/M cell cycle arrest. Most CDTs are hetero-tripartite assemblies of CdtA, CdtB, and CdtC, with CdtB required for CDT-mediated cell cycle arrest. Several lines of evidence indicate that CdtA and CdtC are required for the optimal intracellular activity of CdtB, although the exact functional roles of CdtA and CdtC remain poorly understood. The genes encoding the CDTs have been identified in a diverse array of Gram-negative pathogenic bacteria. More recently, the genes encoding several CdtB subunits have been associated with alternatively linked subunits resembling the B-subunits of pertussis toxin. Although the CDTs are generally considered to all function as bacterial genotoxins, the extent to which individual members of the CDTs employ similar mechanisms of cell surface binding, uptake, and trafficking within sensitive cells is poorly understood. Recently, data have begun to emerge suggesting differences in the molecular basis by which individual CDTs interact with and enter host cells, suggesting the possibility that CDTs possess properties reflecting the specific niches idiosyncratic to those CDT bacterial pathogens that produce them. The extent to which functional differences between individual CDTs reflect the specific requirements for intoxicating cells and tissues within the diverse range of host microenvironments colonized by CDT-producing pathogenic bacteria remains to be experimentally explored.

## Preface

Pathogenic bacteria have acquired attributes that promote adaptation to the specific and often diverse environments encountered within the host. Successful pathogens often actively alter, or remodel, host cells and tissues in order to create a more suitable niche for colonization, often through the direct action of bacterial protein toxins and effectors (Blanke, 2006).

A diverse group of Gram-negative pathogenic bacteria target and modulate the eukaryotic cell cycle through the action of the cytolethal distending toxins (CDTs). Several lines of evidence support the idea that CDTs functions as genotoxins by inducing DNA lesions that ultimately result in cell cycle arrest and cytotoxicity. Current models for both the mechanisms underlying CDT action, as well as the cellular responses to intoxication, have been derived primarily from the study of CDTs from just several toxin-producing species of pathogenic bacteria. However, the discovery of broad carriage of the genes encoding CDTs across many Gram-negative bacteria, as well as the sequence variance between CDTs challenges the notion that individual members within this broad family of these toxins each bind, enter, and deliver their toxic moiety into host cells by an identical mechanism.

## Discovery of the CDTs

The CDTs were discovered approximately 25 years ago as heat labile factors within culture filtrates of enterotoxigenic *E. coli* (Johnson and Lior, 1988a) and *Campylobacter spp*. (Johnson and Lior, 1988b) that induced dramatic and progressive distention of many, but not all, mammalian cell lines. In the decades that have followed their initial discovery, CDTs have been associated with Gram-negative pathogenic bacteria that occupy a diverse array of host niches, including the urogenital tract and the oral cavity. A variety of Gram-negative pathogenic bacteria produce CDTs, including *Aggregatobacter actinomycetemcomitans* (Aa-CDT), *Campylobacter jejuni* (Cj-CDT), *Escherichia coli* (Ec-CDT), *Haemophilus ducreyi* (Hd-CDT), *Helicobacter hepaticus* (Hh-CDT), and *Shigella dysenteria* (Sd-CDT). But how widely found are the CDTs?

## Evolution of the expanding family of CDTs

**Figure 1:**
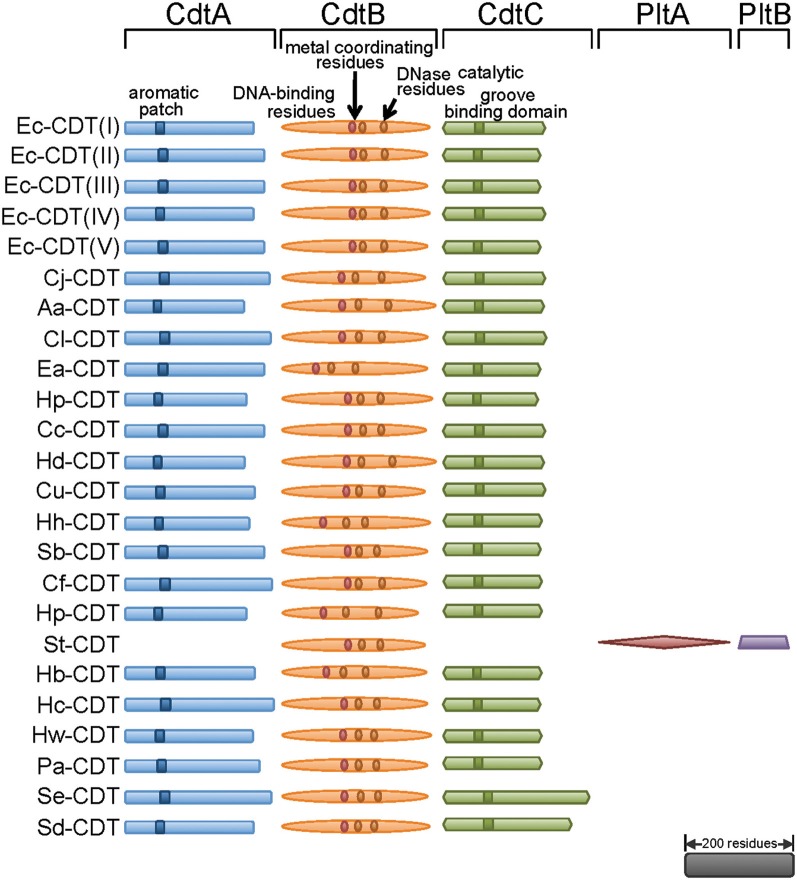
**Three independent proteins are required to make CDT holotoxin.** Most of the known CDTs are composed of CdtA, CdtB, and CdtC, with some exceptions like *S. typhi* where PltA and PltB make holotoxin with CdtB. All CdtAs contain an aromatic patch, demonstrated to be important in binding to the surface of mammalian cells. CdtBs are most conserved amongst three proteins, and are the catalytic subunit of the toxin carrying conserved metal coordinating residues, DNase catalytic residues, and residues homologous to DNA contacting residues of DNase I. CdtCs contain conserved residues believed to be important in formation of a cell surface binding groove. The scale bar represents primary sequence length equivalent to 200 residues. CDT, Cytolethal Distending Toxin; Plt, Pertussis like toxin. Individual genuses and species are abbreviated as follows: Ec, *Escherichia coli*; Cj, *Campylobacter jejuni*; Aa, *Aggregatobacter actinomycetemcomitans*; Cl, *Campylobacter lari*; Ea, *Escherichia albertii*; Hp, *Haemophilus parasuis*; Cc, *Campylobacter coli*; Hd, *Haemophilus ducreyi*; Cu, *Campylobacter upsaliensis*; Hh, *Helicobacter hepaticus*; Sb, *Shigella boydii*; Cf, *Campylobacter fetus*; Hp, *Helicobacter pullorum*; St, *Salmonella typhi*; Hb, *Helicobacter bilis*; Hc, *Helicobacter cinaedi*; Hw, *Helicobacter winghamensis*; Pa, *Providencia alcalifaciens*; Se, *Salmonella enterica*; Sd, *Shigella dysenteriae*.

**Figure 2:**
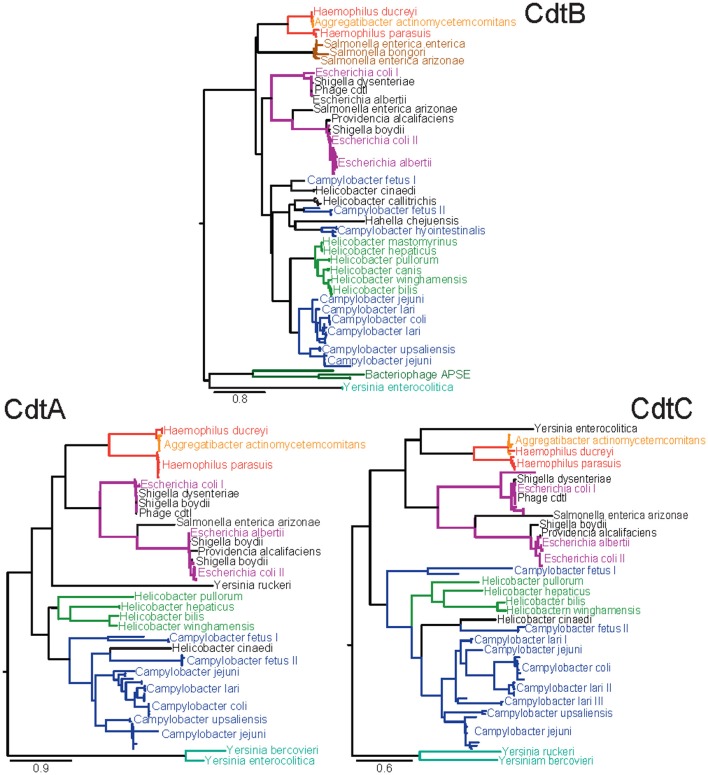
**Amino acid maximum likelihood phylogenies of the three subunits of CDT.** Sequences were extracted from the NCBI non-redundant protein database using BLAST with the criteria that sequences have at least 30% identity and 50% coverage against a known set of CDT subunit sequences from *H. ducreyi, A. actinomycetemcomitans, E. coli, E. albertii, S. dysenteriae, S. enterica, C. jejuni, and H. bilus*. Sequences below these threshold cut-offs were too divergent to be reliably aligned for phylogenetic analyses. Multiple identical sequences from the same species were reduced to unique representatives. The sequences were aligned using a combination of MAFFT and MUSCLE. Phylogenies were inferred using PhyML according to the LG substitution matrix. Clades are colored according to the dominant genus with likely horizontal gene transfer candidates in black. Clades dominated by sequences from a single species are labeled once and distinct clades of sequences from the same species are numbered.

From the mid 1990s and into the early 2000s, the methods for identifying CDT-producing Gram-negative pathogenic bacteria morphed from primarily phenotypic observations of toxin-mediated distention of cultured mammalian cells to genotyping strains for the presence of the genes encoding CDT. The true molecular nature of CDT was revealed from sequencing CDT-encoding regions from enteropathogenic *E. coli* (EPEC) (Scott and Kaper, 1994) and enterotoxigenic *E. coli* (ETEC) (Pickett et al., 1994), which revealed the presence of three open reading frames (designated *cdtA*, *cdtB*, and *cdtC*) organized within a 3-gene operon (Figure 1). Although, widely conserved among CDT-producing pathogens, exceptions to the overall *cdtA*/*cdtB*/*cdtC* gene structure have been identified. Most notably, a distinct CDT with *cdtB* conserved, but having *cdtA* and *cdtC* substituted with genes encoding two homologs of the pertussis toxin (Figure 2) has been described in *Salmonella enterica* serovar Typhi (*S. typhi*) (Spanò et al., 2008), and will be considered separately below.

## Phylogenetic analysis of CDT subfamilies

To obtain insights into the evolutionary and functional relationships between the proteins encoded by these genes, we examined the phylogenetic relationships between the CDT family members using an expanded set of sequences from the NCBI non-redundant protein database, which currently (accessed February 23, 2012) contains 690, 584, and 695 protein sequences annotated to encode CdtA, CdtB, and CdtC, respectively. Phylogenetic analysis revealed a high level of diversity among individual *cdt* genes (Figure 2), consistent with a previous published phylogenetic analysis (Degnan and Moran, 2008). Collectively, 32 species of bacteria and 2 bacteriophages were identified to have genes annotated to encode at least one CDT subunit. Interestingly, the *cdt* genes identified from the current database were restricted to members of the γ-Proteobacteria and ε-Proteobacteria, consistent with earlier reported results (Degnan and Moran, 2008).

The individual phylogenies for each subunit of CDT resolve similar relationships indicating that evolution has occurred largely in the form of vertical descent but is punctuated with occasional instances of horizontal gene transfer adding complexity to the evolutionary history (Ochman et al., 2000). The presence of CDT subunits on bacteriophage further supports this proposal of lateral gene transfer events.

Examination of the phylogenies (Figure 2) reveals that the species of the genus *Yersinia* possess the most unique sequence types of CDT found in the NCBI databases. The branch leading to a *Yersinia* species was the longest branch in each of the three phylogenies indicating that this branch may represent the most ancestral form, or, that evolution of CDT has accelerated along the *Yersinia* lineage (Aguileta et al., 2009). If *Yersinia* does represent the most ancestral form of CDT, these phylogenetic analyses would indicate that CDT originated in the γ-Proteobacteria and was later horizontally transferred into the ε-Proteobacteria based on the evolutionary relationships seen in 16S rRNA phylogenies (Ciccarelli et al., 2006; Yarza et al., 2010). The factors that restrict transfer of genes encoding the CDTs to only species of the γ-Proteobacteria and the ε-Proteobacteria are not clear.

Inconsistencies between the phylogenies for each subunit of CDT are most likely the result of instances of horizontal gene transfer. The species of *Yersinia* represent the most apparent examples. In the case of *Y. ruckeri*, CdtA shows more similarity to the more recently diverged *Haemophilus*/*Aggregatibacter* and *Escherichia* dominated clades. In *Y. enterocolitica*, CdtC shows a closer relationship to the *Haemophilus*/*Aggregatibacter* clade than to other *Yersinia* sequences. As a member of the γ-Proteobacteria, the CdtB sequence of *H. chejuensis* is likely a result of horizontal gene transfer. *H. cinaedi* represents an example of horizontal gene transfer between two closely related genera. Phylogenetically, CDT from *H. cinaedi* is more similar to the *C. fetus* II clade than the *Helicobacter* clade. Although *H. cinaedi* was recently reclassified as a *Helicobacter* species after initial classification as a *Campylobacter* species (Vandamme et al., 1991), it would be expected to be most similar to *H. bilis* based on the 16S rRNA phylogeny (Solnick and Vandamme, 2001). An interesting example of horizontal gene transfer within only one of the three CDT genes was found for *C. lari*, which is broken into three distinct clades for CdtC, while only a single clade with one outlier is observed in both CdtA and CdtB.

Although absent from the CdtB phylogeny (Figure 2), both *Y. ruckeri* and *Y. bercovieri* possess a homolog to CdtB; however, these *Y. ruckeri* and *Y. bercovieri* sequences shared only 30% identity with *Y. enterocolitica* CdtB and were far below the cut-offs used when searching the NCBI database with a reference set of CDT sequences. The published genome of *Hahella chejuensis* revealed the presence of highly divergent CdtA and CdtB subunits that were most similar to those found in the *Yersinia*, but these proteins were also excluded from the phylogenetic analyses due to unreliability of their alignment to other CDT sequences. For *Helicobacter mastomyrinus* and *H. callitrichis*, no sequenced version of CdtA or CdtC was available. Sequences with similarities to CdtA or CdtC were identified within the carboxyla-termini of several *Pseudomonas entomophila* open reading frames. However, these proteins also contained regions of greater than a hundred additional residues lacking sequence similarity to either CdtA or CdtC, and these sequences were excluded from the phylogenetic analyses.

The CdtB sequence has also been detected in multiple variants of the APSE bacteriophage that apparently lack the genes encoding either CdtA or CdtC (Moran et al., 2005). APSE are lysogenic phages that have been found to infect “*Canditatus* Hamiltonella defensa” which is a facultative endosymbiont of numerous sap-feeding insects. The presence of APSE has been linked with the aphid possessing increased resistance against parasitoid wasps (Oliver et al., 2005). Unlike *S. enterica* Typhi, no alternative receptor-binding subunits have been detected (Degnan and Moran, 2008). While the method of delivery of CdtB originating from APSE remains unknown, it has been proposed that the “*Ca*. Hamiltonella defensa” type two secretion system, type three secretion system, or lysis of intracellular endosymbiont cells may be factors in delivery (Degnan and Moran, 2008). Evolutionarily, the presence of CdtB on APSE phages provides both another example of a vector to facilitate horizontal gene transfer as well as an example of co-option of this toxin by a symbiont to provide a better fitness advantage to its host.

The presence of a *Salmonella* clade in the CdtB phylogeny led to a hypothesis of modularity between CdtB and receptor-binding subunits (Spanò et al., 2008). The CdtB sequences found within that clade do not adhere to the canonical association of CdtA, CdtB, and CdtC found in other species. Instead, these CdtB sequences are found in a small pathogenicity island associated with homologs to the pertussis toxin receptor-binding subunits A and B. Previous studies have hypothesized that these pertussis toxin B-fragment homologs in *S. enterica* Typhi may functionally substitute for CdtA and CdtC found in almost all other species (Spanò et al., 2008). Interestingly, our analyses revealed the substitution of the pertussis toxin B-fragment homologs for the canonical CdtA and CdtC subunits within the published genomes of both *S. enterica arizonae* as well as *S. bongeri*. Additionally, potential homologs to both pertussis subunits were found by BLAST in *E. coli* and *Y. enterocolitica*; however, they were not present in same configuration as is seen in *Salmonella*. Finally, it is noteworthy that *S. enterica arizonae* possess distinct copies of both “types” of CDTs. However, the predicted CdtB protein sequence in the “canonical” *cdtA*/*cdtB*/*cdtC* operon is truncated by over 100 amino acid residues.

Taken together, these phylogenetic analyses reveal the emergence of distinct CDT subfamilies, characterized by a high degree of sequence diversity existing among the three subunits. The extent to which this sequence diversity reflects true functional differences between CDTs is largely unexplored. However, as discussed below, several studies have recently hinted at striking dissimilarities in the potencies of CDTs from several different species, as well as the requirement for distinct host determinants required for intoxication, suggesting that individual CDTs may bind, enter, and traffic within host cells in fundamentally different ways.

## Structure and function of CDTs

### Do CDTs possess an AB-toxin architecture?

Intracellular-acting bacterial protein toxins generally possess a two-component “AB” functional architecture (Blanke, 2006). The B components facilitate toxin binding and uptake into sensitive host cells, whereas the A components, which are typically enzymes, act upon specific intracellular targets in a manner that alters or disrupts normal cellular function. Several subclasses of AB toxins can be differentiated based on the number and/or arrangement of the A and B subunits. Diphtheria toxin and exotoxin A from *Pseudomonas aeruginosa* are single-chain AB toxins, whose A and B components are encoded within a single polypeptide that are ultimately separated following proteolysis and reduction of a bridging disulfide linkage. Cholera and pertussis toxins are members of the AB_5_ subclass, characterized by either homo- or hetero-B-fragment pentamers that assemble non-covalently with the catalytic A fragments prior to secretion. Anthrax toxin is a member of a third AB toxin subclass called the binary toxins, comprising separately secreted A and B polypeptides that assemble on the surface of mammalian cells. As discussed below, CDTs may comprise a 4th subclass of AB intracellular-acting toxins.

### Subunit requirements for activity

Several studies have indicated that CdtA, CdtB, and CdtC assemble into a heterotrimeric complex (Lara-Tejero and Galan, 2001; Saiki et al., 2001, 2004; Wising et al., 2002; Nesiæ et al., 2004; Shenker et al., 2004; Cao et al., 2005, 2008; Nesic and Stebbins, 2005; Hu and Stebbins, 2006; Yamada et al., 2006). The maximal potency of CDTs on mammalian cells is generally believed to require the involvement of all three subunits. In fact, several studies have reported that the presence of CdtA, CdtB, and CdtC are absolutely required for Hd-CDT (Lara-Tejero and Galan, 2001; Saiki et al., 2001), Aa-CDT (Wising et al., 2002; Nesiæ et al., 2004; Shenker et al., 2004), and Cj-CDT (Saiki et al., 2004; Cao et al., 2005; Nesic and Stebbins, 2005) to induce cell cycle arrest within intoxicated cells.

In contrast, several studies have indicated that CdtA is not absolutely required for CDT activity. Mixtures of Aa-CdtB/Aa-CdtC retained the capacity to induce G_2_/M cell cycle arrest in the absence of Aa-CdtA (Cao et al., 2005, 2008; Hu and Stebbins, 2006; Yamada et al., 2006). Similarly, mixtures of recombinant Hd-CdtB and Hd-CdtC induced cell elongation characteristic of CDT-intoxicated cells, although the activity was enhanced in the presence of periplasmic extracts containing Hd-CdtA (Deng et al., 2001). In another study, whole-cell sonicates produced from mutant strains of *H. ducreyi* lacking the *cdtB* or *cdtC* genes exhibited a loss of cytotoxic activity toward HeLa cells, whereas the sonicate from the *cdtA* mutant retained cytotoxic activity, albeit at a slightly reduced level (Lewis et al., 2001). For Cj-CDT, combinations of recombinant CdtB and CdtC were only approximately 4-times less potent that mixtures of all three subunits in their capacity to induce cell cycle arrest (Lee et al., 2003).

A more thorough understanding of the specific contributions of the CdtA or CdtC subunits to the potency and action of CDT holotoxins will require the systematic comparison of quantitative dose response curves derived from binary combinations of CDT subunits versus those derived from the corresponding heterotrimeric toxin assemblies.

### Evidence that CdtB is the intracellular-active fragment of CDT

The first indications that CDTs function as intracellular-acting toxins were studies reporting that the cell cycle modulatory activity of Hd-CDT is inhibited when clathrin-mediated endocytosis is blocked (Cortes-Bratti et al., 2000), suggesting that the toxin must be internalized from the cell surface into cells to induce G_2_/M cell cycle arrest. Additional evidence for an intracellular site of action came from transient expression or microinjection (Lara-Tejero and Galan, 2000) of Cj-CdtB, or electroporation (Elwell et al., 2001) of Ec-CdtB alone in mammalian cells, suggesting the enzymatic activity resides within the CdtB subunit of the holotoxin. Moreover, expression of Cj-CdtB alone in yeast also induced G_2_/M arrest (Hassane et al., 2001), analogous to what happens in mammalian cells. Using *in vitro* DNA degradation assays, two groups demonstrated independently that CdtBs from either *E. coli* and *C. jejuni* possess DNase I activity (Elwell and Dreyfus, 2000; Lara-Tejero and Galan, 2000). Mutations in CdtB residues predicted to be functionally equivalent to those in type I DNases required for catalysis or magnesium-binding ablated DNase activity (Elwell and Dreyfus, 2000; Lara-Tejero and Galan, 2000).

Interestingly, several studies have reported intracellular toxin activity associated with the CdtC subunit. CdtC was necessary for G_2_ arrest in human T cells that had been exposed to Aa-CDT with a defective mutant form of the CdtB subunit (Shenker et al., 2000). In addition, protein transfection of CHO cells with recombinant forms of either Aa-CdtB or Aa-CdtC alone induced cellular elongation and cell death (Mao and DiRienzo, 2002).

### The nucleus as the site of CDT action

The capacity of CDT to induce cellular responses normally associated with DNA damage suggests that the ultimate site of CdtB action is the nucleus. In support of this model, the introduction of Ec-CdtB directly into mammalian cells by either electroporation or ectopic overexpression revealed enrichment of CdtB within the nuclear region and G_2_/M cell cycle arrest (McSweeney and Dreyfus, 2004). Mutant forms of the Ec-CdtB lacking putative nuclear localization signals were deficient in nuclear localization and the capacity to induce G_2_/M arrest (McSweeney and Dreyfus, 2004). For Aa-CdtB, deletion of a non-canonical amino-terminal nuclear localization sequence prevented intoxication (Nishikubo et al., 2003). Nonetheless, the capacity of the CdtB subunit to localize to the nucleus of cells that have taken up holotoxin from the cell surface remains to be demonstrated.

### CdtB as a DNase?

The discovery of Cj-CdtB (Lara-Tejero and Galan, 2000) and Ec-CdtB (Elwell and Dreyfus, 2000) catalytic residues conserved with eukaryotic DNase I, first suggested that this CDT subunit might function as an intracellular-acting DNase. High resolution structural data support this idea, as structures for Hd-CdtB (Nesiæ et al., 2004), Aa-CDT (Yamada et al., 2006), and Ec-CdtB (Hontz et al., 2006) possess the canonical four-layered fold of the DNase I family, which is a central 12-stranded β-sandwich packed between outer α-helices and loops on each side of the sandwich. *In vitro* DNase I activity, albeit relatively poor, has been demonstrated for purified CdtB subunits from several bacteria (Elwell and Dreyfus, 2000). Directed mutagenesis of CdtB active site residues predicted by sequence homology to be important for the catalytic activity or the Mg^2+^-binding resulted in inactive forms of CdtB deficient in DNA cleavage activity and in the capacity to induce DNA damage responses leading to cell cycle arrest (Elwell and Dreyfus, 2000; Lara-Tejero and Galan, 2000; Hassane et al., 2001; Guerra et al., 2005). In yeast, ectopic expression of Cj-CdtB was demonstrated to induce homologous recombination and activation of DNA damage checkpoints, which are characteristic mechanisms of cellular responses to DNA damage (Kitagawa et al., 2007).

### CDT as a eukaryotic-like phosphatase?

There is not universal agreement that the cellular effects associated with CDT intoxication are due to the DNA degrading activity of the CdtB subunit. Indeed, sequence homology of CdtBs with members of a broad metalloenzyme superfamily led to the proposal that CdtB induces cell cycle arrest as a phosphatase rather than a DNase (Dlakic, 2001). Support for this hypothesis emerged from studies reporting that *in vitro*, Aa-CdtB demonstrates PI-3,4,5-triphosphate phosphatase activity (Shenker et al., 2007). Mutant proteins with altered residues known to be important for phosphatase activity ablated the *in vitro* activity of Aa-CdtB, as well as the capacity of Aa-holotoxin to induce G_2_/M arrest in human lymphocytes.

To date, phosphatase activity has not been reported for any CDT other than Aa-CDT. Several studies have reported data suggesting that at least some of the cellular consequences of Aa-CDT cellular intoxication are not likely due to phosphatase activity of Aa-CdtB. Ectopic expression of Aa-CdtB in *Saccharomyces cerevisiae*-induced DNA damage and S/G_2_ cell cycle arrest, although yeast does not possess the substrate for the reported Aa-CdtB phosphatidylinositol-3,4,5-triphosphate phosphatase activity (Matangkasombut et al., 2010). Moreover, Aa-CDT-mediated apoptosis in proliferating U937 monocytes was demonstrated to depend solely on the DNase activity of the Aa-CdtB subunit (Rabin et al., 2009). The phosphatidylinositol-3,4,5-triphosphate phosphatase activity is not likely to be idiosyncratic for Aa-CdtB, as this subunit shares a nearly identical sequence to that of the CdtB subunit from *H. ducreyi* (Hd-CdtB).

### Do CdtA and CdtC collaborate as the B fragment of CDT?

The cellular studies cited above strongly support the idea that CdtB functions as the enzymatically active A component of an intracellular-acting toxin. But what about CdtA and CdtC? The canonical role of a toxin's B component is to bind the toxin to the cell surface and facilitate intracellular transport and/or translocation of the catalytic A moiety out of the vesicle trafficking network to access the toxins intracellular target (Blanke, 2006). As discussed above, there are conflicting reports as to the essentiality of CdtA for toxin cellular activity, suggesting that perhaps CdtC functions as the toxin B fragment. Indeed, this notion is supported by independent reports of cellular cytotoxicity associated with CdtB/CdtC alone (albeit requiring higher concentrations than the tripartite holotoxin). However, the sufficiency of CdtB/CdtC for cytolethal distending activity has not been a universal finding. Moreover, reports of cellular cytotoxicity associated with CdtC alone further muddy our understanding of the structure and function of the CDTs.

High-resolution structural data have provided some clues, with the CdtA and CdtC subunits of the Hd-CDT holotoxin bearing structure homology with lectin-like proteins, including the B-fragment repeats of the plant toxin, ricin (Nesiæ et al., 2004). The importance of CdtA and CdtC for toxin binding and uptake from the cell surface has directly been demonstrated in several functional studies (Lee et al., 2003; McSweeney and Dreyfus, 2004, 2005). Fluorescence imaging indicated that Aa-CdtA alone binds on the surface of CHO cells to the same extent as a mixture of all three CDT subunits, and better than Aa-CdtB or Aa-CdtC alone (Mao and DiRienzo, 2002). Cj-CDT was demonstrated to bind to the surface of HeLa cells in a saturable manner (Lee et al., 2003). The CdtA and CdtC subunits both bound to the surface of HeLa cells, while CdtB demonstrated little binding to the cell surface. Using a CELISA, the competition-binding assays revealed that both CdtA and CdtC bound to the surface of cells in a specific manner. However, defining the relative contributions of CdtA and CdtC to binding CDT to the cell surface awaits additional study.

### An unexpected exception to the canonical CdtA/CdtB/CdtC structure

An interesting exception to the CdtA/CdtB/CdtC tripartite structure characteristic of the CDT superfamily was discovered within *S. enterica*, serovar Typhi CDT (St-CDT) (Haghjoo et al., 2004), for which the gene encoding CdtB is not associated with genes resembling *cdtA* or *cdtC*. Instead, the cellular modulatory activity of St-CDT is dependent upon expression of two genes, *pltB* (pertussis-like toxin B) and *pltA* (pertussis-like toxin A) (Figure 1), which encode proteins resembling the B components of pertussis toxin, an unrelated AB_5_-intracellular-acting toxin. The reconstituted CdtB/PltA/PltB tripartite complex was demonstrated to induce DNA damage within intoxicated eukaryotic cells (Spanò et al., 2008). Infection studies revealed that bacterial uptake into host cells triggers expression of *cdtB*/*pltA*/*pltB*, and that the PltB and PltA subunits are required for transport of intracellular St-CDT to the extracellular medium, where St-CDT then acts upon uninfected host cells in a paracrine manner (Spanò et al., 2008). Such a mechanism has not been previously described for any AB-toxin, and suggests an expanded role beyond the canonical functions normally associated with the B components of intracellular-acting toxins.

## Mechanism of CDT cellular intoxication

As intracellular-acting genotoxins, the CDTs have several fundamental problems to solve in order to exert their modulatory activities, with the most glaring being how to transport soluble CdtB moieties from the cell surface to the nucleus. There are some recent indications, as discussed below, that host cell requirements for the genotoxic activity of CDTs from different species may be divergent, raising the possibility that not all CdtB moieties travel from the cell surface to the nucleus along a single transport pathway.

### Do Cdts float into cells on membrane rafts?

Cell surface binding and intoxication by several CDTs have been reported to require toxin binding to cholesterol/sphingomyelin-rich microdomains within the outer leaflet of the plasma membrane called membrane rafts. Aa-CDT (Boesze-Battaglia et al., 2006) binds to GM1-enriched regions of the plasma membrane, which are characteristic of membrane rafts. Cholesterol depletion reduces the ability of both Aa-CDT (Boesze-Battaglia et al., 2006) and Hd-CDT (Guerra et al., 2005) to bind to the surface of sensitive cells, and prevents intoxication. Finally, mammalian cell lines with an inactivated *SGMS1* gene, resulting in reduced levels of the key raft component sphingomyelin, were relatively resistant to Ec-CDT (Carette et al., 2009). Although the functional significance of CDT association with membrane rafts is not fully appreciated, toxin binding may be important for uptake into cells, as one of cellular functions associated with rafts is the clustering of the molecular machinery associated with endocytosis.

### CDT receptors—does one size fit all?

The presence or absence of receptors on the plasma membrane surface often dictates the sensitivity or resistance, respectively, of host cells to specific intracellular-acting bacterial toxins (Blanke, 2006). Using ligand blotting assays, very early studies reported the binding of partially purified Cj-CDT to two peptides with approximate molecular masses of 45 and 59 kDa from HeLa cell membrane preparations and 59 kDa from CHO cell membrane preparations (Bag et al., 1993), suggesting the presence of molecular components on the surface of host cells that might function as CDT receptors.

To date, bonafide CDT receptors have not yet been identified. One study implicated fucose as an important determinant for Ec-CDT, as lectins targeting fucose moieties-inhibited toxin interactions with cells to a greater extent than lectins targeting alternative glycans (McSweeney and Dreyfus, 2005). Another study indicated that the Aa-CDT holotoxin binds to surface glycosphingolipids, and that inhibitors of glycosphingolipid synthesis can prevent intoxication of the human monocytic U937 cell line (Mise et al., 2005). Mutagenesis of a human cell line haploid for most chromosomes identified cell lines deficient in the membrane-expressed protein TMEM181 that were resistant to Ec-CDT, although it is not yet clear that TMEM181 functions as a cell surface receptor for this toxin (Carette et al., 2009).

A recent study in which Aa-CDT, Cj-CDT, Ec-CDT, and Hd-CDT were compared in a head to head fashion revealed considerable differences in the potencies of these toxins, as well as the target cell requirements important for intoxication (Eshraghi et al., 2010). For example, cholesterol was found to be important for the cellular activities of Aa-CDT, Cj-CDT, and Hd-CDT, but not for those of Cj-CDT. Using mutant CHO cell lines lacking N-linked complex- or hybrid-carbohydrates, glycosphingolipids, or fucose, the study also reported the unexpected finding that, N- and O-glycan, or fucosylated structures are in fact not required for mediating toxin binding (Eshraghi et al., 2010). The diversity of cell surface determinants important for CDT interactions identified in these early studies suggest that individual CDTs may each bind to a different receptor. It is attractive to speculate that CDT receptor diversity reflects the tissue tropism of individual CDT-producing pathogens.

### Riding the rail: CDT uptake into host cells

Receptor-binding not only concentrates toxins on the surface of host cells, but also positions bound toxin molecules to exploit existing cellular mechanisms for uptake of proteins into cells (Blanke, 2006). Several studies indicate that CDTs are rapidly taken up from the cell surface into an intracellular compartment, suggesting that these toxins are internalized by an endocytic-like mechanism. In support of this idea, Hd-CDT-mediated cell cycle arrest was demonstrated to be dependent on dynamin (Cortes-Bratti et al., 2000), which contributes to several overall mechanisms of endocytosis.

### Retrograde trafficking to the endoplasmic reticulum (ER)

**Figure 3:**
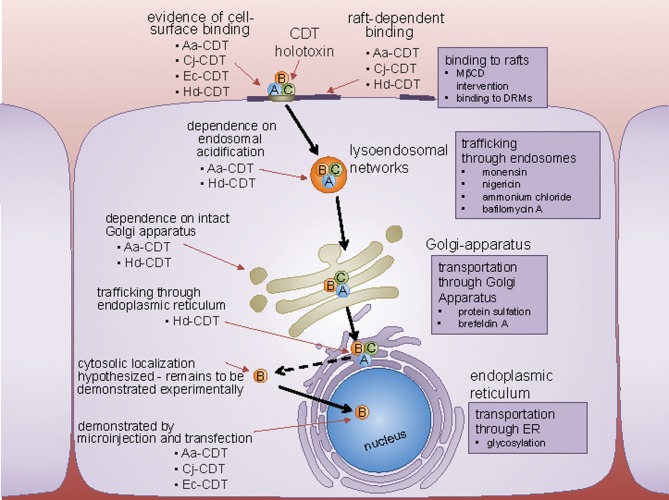
**Host cell surface binding and internalization of CDT.** CDT binds to a cell surface determinant, and in some cases, is affected by perturbations in lipid rafts. Following internalization by clathrin-coated pits to the endosomes, CDT is trafficked in a retrograde fashion to the Golgi-apparatus, and the endoplasmic reticulum (ER). From the ER, CdtB localizes to the nucleus by a yet unconfirmed route directly, or through the cytoplasm. Experimental techniques used to identify the pathway employed by CDT to trafficking inside the cell, are represented next to the organelles involved.

For CDT to function as a genotoxin, the CdtB subunit must escape degradation within the endocytic trafficking pathway and localize to the nucleus. Biochemical and fluorescence imaging studies suggest that CDTs are transported in a retrograde manner from the endo-lysosomal system to the ER via the Golgi complex (Guerra et al., 2005) (Figure 3). Analogous to several other toxins, including cholera and Shiga toxins, the catalytically active subunit (CdtB) is believed to be translocated from the ER to the cytosol by exploiting the ER degradation pathway, which normally translocates misfolded proteins to the cytosol via protein translocons within the ER membrane (Blanke, 2006). One study reported that Hd-CdtB was not detected within the cytosol of intoxicated cells (Guerra et al., 2009). In fact, to date, there is no experimental evidence that any CDT subunit is translocated from the ER to the cytosol of intoxicated cells, leading to the suggestion that the CdtB subunits may be translocated directly from the ER to the nucleus (Guerra et al., 2009).

Clearly, a major gap in the mechanism underlying CDT-mediated cellular intoxication is how CdtB reaches its site of action. As discussed above, the putative nuclear localization signals identified for two members of the CDT family, Aa-CDT and Ec-CDT, are highly divergent, suggesting that the mechanisms by which these two toxins are transported to the nucleus are not necessarily identical.

## Modulation of cellular signaling within CDT-intoxicated cells

The designation of CDT as a genotoxin derives from the demonstration that isolated CdtB (1) functions as a DNase *in vitro* (Elwell and Dreyfus, 2000; Lara-Tejero and Galan, 2000), (2) induces nuclear fragmentation and chromatin disruption when transfected in cultured mammalian cells or *Saccharomyces cerevisae* (Hassane et al., 2001), and, (3) promotes DNA fragmentation in cells intoxicated with exogenously added CDT (Frisan et al., 2003). CDT-mediated insults to genome integrity induces cellular responses geared toward the repair of DNA lesions and cell survival.

### Activation of DNA repair responses

Most (yet not all) of the experimental evidence to date suggests that the cellular modulatory activity of CDTs originates from toxin-mediated DNA damage, which activates the cellular DNA repair machinery. CDT-dependent DNA damage induces the recruitment of the DNA damage sensor complex MRN and the ATM kinase, which phosphorylates H2AX, a member of the histone 2A family, and activation of several DNA damage checkpoint responses. Several studies have demonstrated that activation of kinase CHK2 leads to inactivation of the CDC25 phosphatase, resulting in the accumulation of the hyperphosphorylated (and inactive) form of the cyclin-dependent kinase CDK1 (pCDK1), leading ultimately to arrest at the G_2_/M interface (Sert et al., 1999; Cortes-Bratti et al., 2001; Li et al., 2002; Sato et al., 2002; Yamamoto et al., 2004; Cuevas-Ramos et al., 2010), and in some cases, transition into a senescent state (Blazkova et al., 2010). A second consequence of CDT-dependent DNA damage is activation of the tumor suppressor p53 and its downstream effector p21, leading to cell cycle arrest at the G_1_/S checkpoint in this case.

### Cashing it in? apoptosis in CDT-intoxicated cells

CDT-dependent apoptosis has been reported in many cell types. In general, CDT intoxication of cells of non-haematopoietic origin results in cell cycle arrest preceding apoptosis (Jinadasa et al., 2011). In contrast, CDT intoxication of haematopoietic cells results in rapid apoptosis, indicating differential cellular responses to the action of CDTs.

### Activation of cell survival responses

While CDT-intoxicated cells have been demonstrated to undergo apoptosis, there are reports that in some cases, CDTs also mobilize cell survival pathways. The survival of CDT intoxicated cells is dependent on the activation of the small GTPase RhoA (Frisan et al., 2003), which induces actin stress fiber formation, and prevents cell death via activation of the mitogen-activated protein kinase p38 and its downstream target mitogen-activated protein kinase-activated protein kinase 2 (Guerra et al., 2008). The activation of RhoA is dependent on the dephosphorylation of the RhoA-specific guanine nucleotide exchange factor Net1, and it appears to be part of the DNA damage repair responses because it requires a functional ATM (Guerra et al., 2008).

## Could the genotoxic action of CDT increase the risk for cancer within the host?

Factors that disrupt normal cell cycle progression may promote acquisition of the cancerous phenotype. While CDT clearly is a modulator of the cell cycle, does the action of this genotoxin during infection contribute to the development of a cancerous phenotype?

### Development of pathogen-mediated inflammatory microenvironments

Infection with pathogenic bacteria has increasingly been associated with malignant disease in humans. However, when considering putative risk factors for cancer, the leap from association to causality can be perilous. How might persistent bacterial infection increase cancer risk? The evolution of pre-malignant to cancerous cells is often characterized by a “mutator phenotype” in which mutations accumulate at an accelerated rate, resulting in genomic instability (Raptis and Bapat, 2006). One of the ways that bacterial infections are thought to promote acquisition of the mutator phenotype is the establishment of a chronic pro-inflammatory microenvironment.

How might persistent bacterial infections induce inflammatory microenvironments? During chronic infection, host tissues are constantly exposed to bacterial PAMPs, which are sensed by pattern recognition receptors (e.g., Toll-like receptors) (Grivennikov et al., 2010). Sustained activation of pattern recognition receptors results in elevated levels of pro-inflammatory cytokines, as well as reactive oxygen species (ROS) and reactive nitrogen intermediates (RNI), which are genotoxic agents that increase the rate and incidence of mutations (Vogelmann and Amieva, 2007).

The most extensively studied association of pathogen-dependent establishment of pro-inflammatory microenvironments and cancer is the increased risk of gastric adenocarcinoma in those individuals infected with *H. pylori* (Polk and Peek, 2010). Notably, chronic *H. pylori* infection correlate with a significant (5-fold) increase in mutation frequency and microsatellite instabilities within the gastric epithelium of infected mice (Touati et al., 2003). Other examples are chronic carriage of *S. enterica*, serovar Typhi, and *Bacteroides fragilis*, which have been associated with gallbladder and colon cancers, respectively (Toprak et al., 2006; Vogelmann and Amieva, 2007). Moreover, *Streptococcus bovis* with colon cancer, *Chlamydia pneumoniae* with lung cancer, and *Bartonella* species with vascular tumor formation (Vogelmann and Amieva, 2007).

### Whence CDTs and malignancies?

The contributions, if any, of CDTs to the development of human malignancies are largely unexplored. Analogous to genotoxic agents such as ROS and RNI that increase the rate of mutations within the genome (Karin et al., 2006; Grivennikov et al., 2010), one possibility is that CDT might contribute to progressive genome destabilization within the pathogen-induced pro-inflammatory microenvironments (Grivennikov et al., 2010). CDT activation of survival responses in cells harboring DNA damage could contribute to the accumulation of genetic instability within the developing inflammatory micro-environment, which may constitute the perfect storm of conditions for promoting the transformation of pre-neoplastic cells to malignant cells in the host.

Several studies have begun to address whether infection with CDT-producing pathogenic bacteria may induce changes in the host associated with an early neoplastic process. Using NF-κB-deficient mice to study persistent *C. jejuni* infection, one study indicated a proinflammatory role for Cj-CDT *in vivo*, as toxin-producing strains were associated with significantly enhanced severity of gastritis and significantly higher occurrence of gastric hyperplasia and dysplasia, which are markers of an early neoplastic process (Fox et al., 2004). For *Helicobacter hepaticus*, which causes chronic hepatitis and typhlocolitis in some mice, CDT was demonstrated to be critical for persistent infection of Swiss Webster mice, which is characterized with severe inflammation (Ge et al., 2005). In another study, *H. hepaticus* infection of A/JCr mice demonstrated a significantly higher inflammatory response in strains producing Hh-CDT than isogenic mutant strains. (Ge et al., 2007). The presence of CDT was associated with a progression of inflammation to dysplasia. In the future, it will be important to conduct studies that will evaluate potential associations between CDT-dependent dysplasia and toxin-mediated DNA damage, chronic induction of DNA damage repair pathways, and the acquisition of the type of genomic instability preceding acquisition of the mutator phenotype discussed above.

## On to the future …

One cannot help but be fascinated by the vertical transmission of phenotypic traits within families that can result in strikingly similar appearance and mannerisms. But, as is often the case when we dig below the surface, interesting and important differences emerge which define the unique identity of individual family members.

When considering the “CDT family,” a natural focus has centered on the similarities among the individual members, and to figure out, in essence, how they “look alike.” Most of the excellent work that has been conducted thus far, by many different laboratories, to identify and characterize the structure-function relationships and intoxication properties has appropriately focused on just several CDTs (e.g., Aa-CDT, Cj-CDT, Ec-CDT, and Hd-CDT). The conclusions from studying this small subset of the CDT family have been extended rather generically across the broader family of CDTs. Yet, neither the structure-function relationships nor the mechanisms underlying cellular intoxication of cells by the vast majority of CDTs have been examined. In fact, the striking diversity in the sequences of CDT subunits identified in an increasing number of genomes of bacterial species from the γ-Proteobacteria and ε-Proteobacteria suggest that the extent to which individual CDTs function in a similar fashion should be reexamined.

The degree to which the large sequence diversity among CDTs reflects functional differences between individual members of the CDT family is largely unexplored. Nonetheless, as discussed above, there are some hints from several recent studies that CDTs may in fact be highly divergent in their potencies and specific host requirements for intoxication. It will be interesting to test the premise that the functional differences between CDTs may reflect the diversity of host cells and tissues that compromise the infection microenvironments targeted by CDT-producing pathogenic bacteria.

One of the true challenges in the CDT field is to understand the *in vivo* consequences of CDT action during infection. Studies to understand, from an evolutionary perspective, the benefit of CDT-mediated genotoxicity for promoting host-pathogen interactions, remain a particular challenge, because any benefits may in fact be highly idiosyncratic to the specific pathogen and the host niche occupied by that pathogen.

### Conflict of interest statement

The authors declare that the research was conducted in the absence of any commercial or financial relationships that could be construed as a potential conflict of interest.
